# Predicting energy prices and renewable energy adoption through an optimized tree-based learning framework with explainable artificial intelligence

**DOI:** 10.1038/s41598-026-35706-z

**Published:** 2026-01-30

**Authors:** Tao Tang

**Affiliations:** Institute of Xi Jinping Thought on Socialism with Chinese Characteristics for a New Era, Peking University, Beijing, China

**Keywords:** Global energy consumption, Energy price index, Renewable energy share, Sensitivity analyses, Feature importance, Energy science and technology, Engineering, Environmental sciences, Mathematics and computing

## Abstract

This research offers a comprehensive analysis of global energy consumption, focusing on predicting two key metrics: the Energy Price Index and the Renewable Energy Share. The study employs advanced Machine Learning (ML) regression techniques, all further optimized using metaheuristic algorithms. In addition, a primary objective of this study is to determine which variables most significantly affect model performance and predictive accuracy. Through SHAP (SHapley Additive exPlanations) and CAM (Cosine Amplitude Method) sensitivity analyses, the study systematically interprets model outputs and quantifies the influence of each input feature. Findings demonstrate that, according to the SHAP-based model interpretation, the prediction of Renewable Energy Share is most strongly influenced by fossil fuel dependency and carbon emissions. These results underscore the pivotal role of consumption intensity and environmental indicators in shaping both global energy price trajectories and renewable energy adoption rates. Integrating optimization algorithms with advanced models improved both predictive accuracy and model robustness. The resulting analytical framework provides a technically rigorous and interpretable approach to global energy forecasting. Such a framework is valuable for informing energy policy, supporting sustainability strategies, and enabling stakeholders to monitor environmental impacts and optimize energy system performance. By leveraging data-driven insights, this study advances practical tools and methodologies for strategic planning in the context of a sustainable global energy future.

## Introduction

Global energy consumption has been increasing rapidly due to population growth, industrialization, and urbanization, driving significant fluctuations in energy prices and raising the demand for sustainable energy solutions. Despite efforts to diversify energy sources, fossil fuels continue to dominate the global energy mix, contributing substantially to greenhouse gas (GHG) emissions and environmental degradation^1,2^. Understanding the dynamics of energy demand and supply, along with the factors influencing energy price volatility, is therefore essential for global sustainability and economic planning. Past studies have quantified the environmental and economic impacts of energy consumption, focusing on greenhouse gas emissions from industrial, residential, and transportation sectors^3,4^.

Energy demand intensity, fossil fuel dependency, and regional consumption patterns are critical drivers of price fluctuations, particularly in areas with high per-capita energy use. While energy and industrial systems have been the primary focus of GHG mitigation strategies, there is a growing need to integrate predictive analyses of energy prices and renewable energy adoption to support informed policy and market decisions^5–7^. Despite extensive research on energy consumption trends and emissions, there remains a noticeable gap in studies that combine accurate energy price forecasting with the adoption of renewable energy sources using advanced machine learning (ML) models^8,9^. Developing data-driven models that can identify key drivers of energy dynamics and predict future trends is essential for enabling energy market stakeholders to optimize resource allocation, reduce risks, and design effective sustainability strategies.

As a result, little is known about the extent to which irrigation-related GHG emissions contribute to total agricultural GHG emissions and how they affect international efforts to mitigate climate change. Developing effective mitigation methods to achieve NetZero agriculture emissions requires a thorough, globally dispersed examination of the energy consumption and GHG emissions inherent in irrigation and pumping systems^10,11^. Although energy and industrial systems have been the primary focus of efforts to reduce GHG emissions, research on GHG reductions in agriculture, which contributes 12% of global GHG emissions (7.1 Gt CO2 equivalent annually), has received relatively less attention. Furthermore, rather than actively lowering energy use and CO2 emissions, the focus has mostly been on improving irrigation water efficiency^12,13^. The sustainable development of irrigated agriculture is therefore urgently needed to increase food production while reducing dependence on energy, water, and greenhouse gas emissions^14,15^.

Global energy consumption has been rising steadily due to population growth, industrialization, and urbanization, driving significant fluctuations in energy prices and increased demand for sustainable energy solutions. Understanding the dynamics of energy markets, including price volatility and the adoption of renewable energy, has become a central concern for policymakers and industry stakeholders worldwide.

Yang et al. ^16^ proposed a hybrid model combining GA-optimized VMD, ICEEMDAN, BiGRU, TCN, and MLP for multi-step prediction of carbon and natural gas prices. The model decomposes the price series into subsequences, predicts each component separately, and recombines them for final forecasts. Results showed it outperforms traditional models across MAE, RMSE, MAPE, R², and modified Diebold-Mariano tests, especially for short-term predictions. Ukoba et al. ^17^ examined the role of ML in optimizing renewable energy systems and supporting climate change mitigation. It highlighted how ML can enhance efficiency, reliability, and scalability of renewable technologies, as well as improve climate projections and practical mitigation strategies, while noting challenges such as model interpretability, data quality, and ethical considerations.

The World Energy Council^18^ outlines future scenarios with projections of global energy consumption patterns until 2060. The report examines energy consumption by fuel type and end-user sector, highlighting potential shifts toward sustainable energy systems. The report emphasizes that without effective intervention, energy consumption patterns may undermine climate targets, while proactive policy and technological measures could accelerate the transition to low-carbon energy systems. Parallel to these issues is the view of Ahmed and Faroque^19^, whereby they analyzed the composition and environmental effects of global energy consumption. It supports the World Energy Council’s observations that fossil fuels dominate the global energy mix and are a major contributor to greenhouse gas emissions. Their work underscores the importance of renewable energy adoption to mitigate environmental risks and enhance energy system resilience. Hasan and Sadikul^20^ analyzed global energy consumption trends from 2011 to 2023, demonstrating a gradual but consistent transition toward renewable energy sources driven by technological innovation and policy frameworks. These studies collectively illustrate the critical interplay between energy consumption patterns, market stability, and the pace of renewable energy adoption. Alazemi et al. ^21^ reviewed ML approaches for forecasting renewable energy sources (RES) at the distribution level. It highlighted that deep neural networks, particularly LSTM, and ensemble methods effectively handle the stochastic and autoregressive nature of RES outputs, outperforming traditional physical and statistical models. The review also discussed integrating forecasts into decision-making processes, such as unit commitment, to address economic and operational challenges in power grids, and outlines directions for future research.

Accurate forecasting of energy prices and renewable energy shares requires advanced analytical methods capable of capturing non-linear relationships among multiple economic, environmental, and policy variables. Yucesan and Melih^22^ compare artificial neural networks (ANNs) with conventional regression analysis to forecast global energy consumption. Based on the research, ANNs are found to have superior precision, especially when modeling the non-linear relationship between energy consumption and factors that govern it, including population growth. The results identify population as one of the most prominent drivers of energy demand, with models that contain population data outperforming others in predictability. Recent research increasingly applies ML and ensemble-based methods to model energy market dynamics, offering greater precision and robustness than traditional approaches.

Despite progress in ML-based energy forecasting, challenges remain in model interpretability and feature importance assessment. Understanding which variables drive energy price fluctuations and renewable energy adoption is crucial for providing policymakers and industry stakeholders with actionable insights. This study addresses these challenges by integrating optimized tree-based learning frameworks with explainable artificial intelligence techniques, such as SHAP and CAM, to quantify the influence of input features on predictive outcomes.

This research provides accurate forecasts for the Energy Price Index and the Renewable Energy Share, delivering insights that support strategic decision-making, resource allocation, and policy development. By focusing on the most influential drivers of predictive performance, the study enhances model reliability and facilitates data-informed strategies to promote renewable energy adoption and manage energy market volatility. This study offers significant contributions with practical implications for energy market stakeholders. Delivering accurate forecasts of the energy price index and the share of renewables enables informed and strategic decision-making, moving beyond speculative approaches. Such data-driven insights are essential for optimizing resource allocation, guiding investment strategies, and shaping effective policy frameworks in an increasingly dynamic energy landscape. Notably, the research goes beyond surface-level predictions by identifying the key drivers that influence predictive performance. This deeper analytical focus enhances methodological refinement, leading to improved reliability and precision in forecasting outcomes. Rather than emphasizing technological novelty for its own sake, the study underscores the practical value of predictive accuracy in generating actionable intelligence. These insights support the development of more effective energy market strategies and facilitate the broader adoption of renewable energy sources. At a macro level, the findings serve as a valuable resource for policymakers and industry leaders, offering guidance to reduce inefficiencies, mitigate risks, and foster innovation. Overall, this research acts as a catalyst for advancing economic efficiency, environmental sustainability, and the evolution of smarter, data-informed energy systems.

## Data gathering

The Global Energy Consumption stands out as a comprehensive resource, providing two decades of detailed data from a diverse range of countries and sectors. The dataset is invaluable for investigating energy consumption patterns, assessing regional disparities in energy demand, and examining the integration of renewable energy alongside evolving carbon emission trends. Analysts can visualize global and regional energy consumption trends, forecast future demand to guide policy and strategic planning, and evaluate the relative contributions of renewables and fossil fuels within the global energy mix. The dataset al.so supports the exploration of relationships among energy use, economic development, and carbon emissions, thereby informing sustainability initiatives. The dataset consists of country–year observations derived from a global energy database covering the period 2000–2024. Each record corresponds to a single country in a given year. The dataset, referred to as “Global Energy Consumption Dataset”, is sourced from^23^. Data were collected across more than 50 countries, yielding 10,000 country–year observations after data integration and preprocessing. The final sample size reflects the aggregation of multiple countries across multiple years, rather than a single annual time series. All variables, including the Energy Price Index and Renewable Energy Share, are reported on an annual basis at the country level. This structure allows the models to learn both temporal patterns and cross-country heterogeneity in global energy dynamics.

The accompanying statistical summary presents key features relevant to energy systems analysis in Table 1. The six input variables include Per Capita Energy Use (kWh), Fossil Fuel Dependency (%), Industrial Energy Use (%), Household Energy Use (%), Total Energy Consumption (TWh), and Carbon Emissions (Million Tons). Two output variables, Energy Price Index (USD/kWh) and Renewable Energy Share (%), are also provided. For each, the dataset reports maximum, minimum, mean, median, standard deviation, and skewness. Per Capita Energy Use varies widely, from approximately 500 to nearly 50,000 kWh, with a mean and median around 25,000 and minimal skewness, indicating a fairly symmetric distribution. Fossil Fuel Dependency ranges from 10% to 80%, with most values clustering near the mean of 45%. Both Industrial and Household Energy Use show moderate variability and near-zero skewness. Total Energy Consumption and Carbon Emissions demonstrate substantial dispersion, yet their distributions remain close to symmetric. The Energy Price Index ranges from $0.05 to $0.50 per kWh, with moderate variability and slight positive skewness. Renewable Energy Share is highly variable, spanning from 5% to 90%, but the distribution remains largely symmetric. Overall, the dataset provides a balanced view of global energy consumption and related variables. The distributions are close to symmetric, while the degree of variability varies across features. This breadth and depth of data facilitate rigorous, data-driven analysis and informed decision-making in the context of global energy transitions.

**Table 1:** Overview of input features and output variables with their statistical properties.

*Category*	*Variables*	Unit	Characteristics					
			**Max**	**Min**	**Mean**	**Median**	**St. Dev.**	**Skewness**
***Inputs***	***Per Capita Energy Use***	**(kWh)**	49989.6	500.27	25040.0	25098.8	14204.9	0.015
	***Fossil Fuel Dependency***	**(%)**	80	10.01	44.93	45.11	20.202	-0.003
	***Industrial Energy Use***	**(%)**	60	20	40.06	39.98	11.538	-0.007
	***Household Energy Use***	**(%)**	40	10	25.04	25.09	8.597	-0.020
	***Total Energy Consumption***	**(TWh)**	9999.3	100.48	5142.6	5190.9	2848.61	-0.044
	***Carbon Emissions***	**(Million** **Tons)**	4999.3	50.64	2536.2	2568.0	1424.03	-0.016
***Output***	***Energy Price Index***	**(USD/kWh)**	0.5	0.05	0.27	0.27	0.131	0.009
	***Renewable Energy Share***	**(%)**	90	5	47.32	47.15	24.603	0.014

### Correlation between the variables

**Fig. 1:**
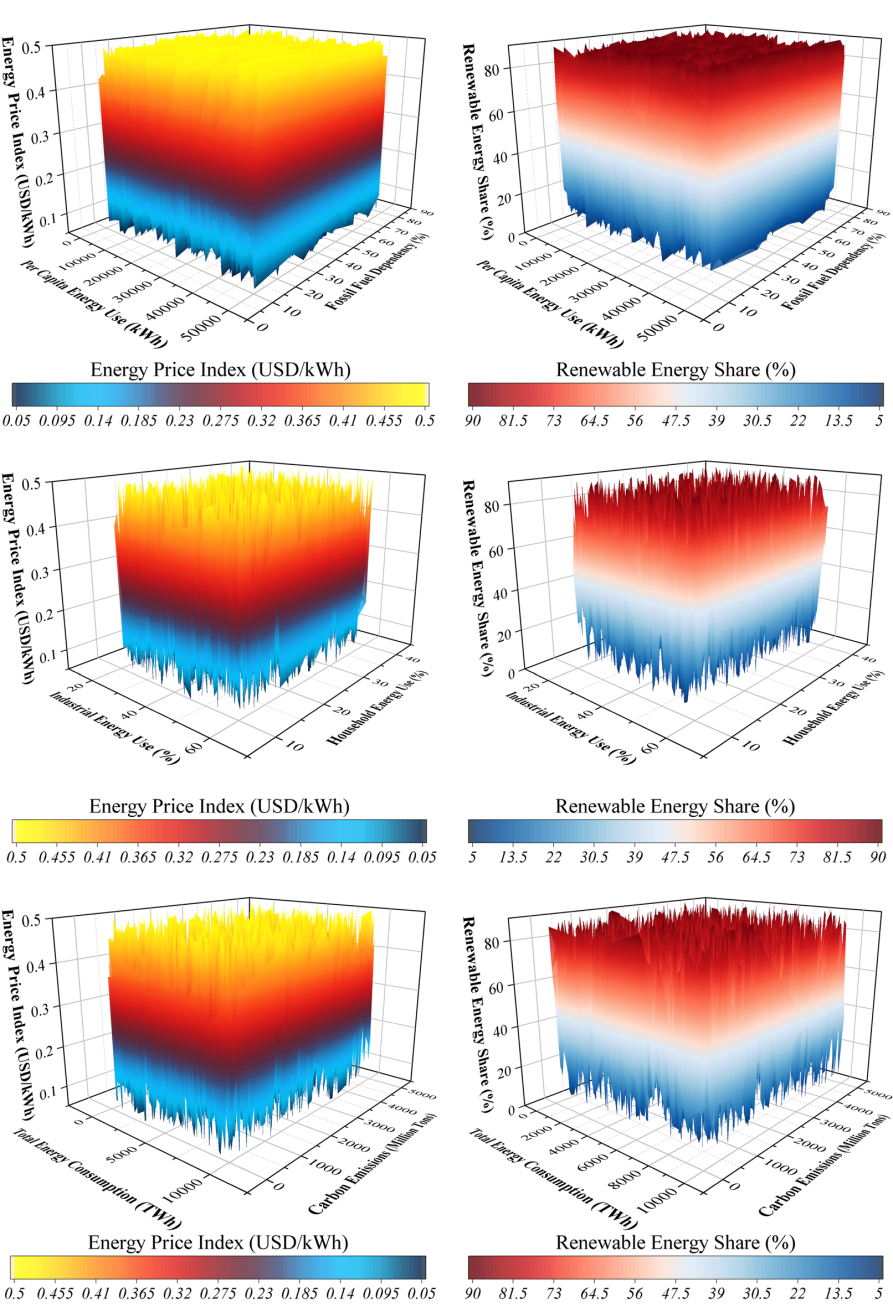
3D surface for the distribution and correlation within the variables across different targets.

Figure 1 presents the distribution and correlations among the input variables for the Energy Price Index (EPI) and Renewable Energy Share (RES). This approach avoids premature feature elimination and allows models to learn nonlinear interactions directly, while correlation analysis provides contextual insight into pairwise relationships. For the Energy Price Index, fossil fuel dependency and per-capita energy use exhibit strong positive correlations with price fluctuations. Countries that rely heavily on coal, oil, and gas experience greater price volatility, particularly during periods of constrained supply. Regions with high per-capita energy consumption, such as North America, are more sensitive to changes in fossil fuel availability.

Regarding Renewable Energy Share, industrial and household energy consumption are highly correlated with renewable adoption. Industrial energy use, which often accounts for 30–60% of total consumption in developed economies, enables economies of scale that reduce renewable energy costs, while household consumption (20–30%) contributes through distributed installations such as rooftop solar. Pearson correlation coefficients range from 0.6 to 0.8, indicating a substantial linear association between these variables and renewable adoption. These correlation insights guided the selection of input features for the optimized tree-based learning framework. By identifying the most influential variables, ML models can capture both linear and nonlinear relationships, and feature importance can be further interpreted using SHAP and CAM analyses.

### K-fold Cross-validation

Table 2 presents the results of the 5-fold cross-validation procedure for the Energy Price Index and Renewable Energy Share targets. The analysis is intended to evaluate the stability and robustness of predictive performance across different data partitions rather than to compare individual learning algorithms. Accordingly, the discussion emphasizes fold-wise trends and performance consistency. Although point estimates are reported, robustness is evaluated through fold-wise consistency under the 5-fold cross-validation framework. The limited variation in R² and RMSE values across folds (K1–K5) indicates that model performance is not driven by a specific data split. This stability across folds provides an empirical measure of robustness without requiring repeated random runs or parametric confidence intervals. For the Energy Price Index, the averaged R² values across all models increase from 0.921 in K1 to 0.938 in K5, indicating progressively improved explanatory capability as the training set expands across folds. A corresponding reduction in the averaged RMSE values is observed, decreasing from 0.049 in K1 to 0.043 in K5. This monotonic improvement across folds highlights stable generalization behavior and limited sensitivity to data partitioning. A similar pattern is evident for the Renewable Energy Share target. The fold-averaged R² values rise steadily from 0.917 (K1) to 0.932 (K5), while the averaged RMSE decreases from 10.91 to 8.73. These results demonstrate consistent learning behavior across folds despite the higher intrinsic variability associated with renewable energy adoption data.

To evaluate the predictive performance and generalization capability of the ML models, a 5-fold cross-validation (CV) procedure was implemented. For each fold, the dataset was split into training (80%), validation (10%), and test (10%) subsets, ensuring that the test set remained unseen during both model training and hyperparameter optimization. Given the temporal nature of the energy price and renewable energy data, care was taken to prevent data leakage. Specifically, folds were constructed using a chronological split, with earlier time periods assigned to training and later periods to validation and testing. This approach preserves temporal causality and avoids artificially inflating model performance. Hyperparameter optimization using COA and OOA was conducted exclusively on the training and validation sets. The best-performing parameters were then applied to the test set to report final metrics, including R² and RMSE. For reproducibility, all experiments were executed in Python 3.10 using the Scikit-learn library (v1.2.2) on a system with 32 GB RAM and an Intel Core i7 processor. Random seeds were fixed (seed = 42) for both model initialization and optimizer procedures, ensuring consistent reproducibility.

**Table 2:** Results produced by the 5-fold cross-validation procedure.

*Target*	*Indicator*	*Model*	Number of K-Folds				
			K1	K2	K3	K4	K5
**Price Index**	**R** ^**2**^	HGBR	0.940	0.944	0.961	0.951	0.958
		ETR	0.920	0.924	0.933	0.930	0.938
		DTR	0.902	0.906	0.915	0.912	0.919
		Average	0.921	0.925	0.936	0.931	**0.938**
	**RMSE**	HGBR	0.054	0.052	0.046	0.050	0.047
		ETR	0.048	0.046	0.044	0.045	0.042
		DTR	0.046	0.044	0.042	0.043	0.040
		Average	0.049	0.047	0.044	0.046	**0.043**
**Renewable Energy**	**R** ^**2**^	HGBR	0.940	0.947	0.953	0.952	0.956
		ETR	0.918	0.924	0.931	0.929	0.934
		DTR	0.892	0.898	0.904	0.903	0.907
		Average	0.917	0.923	0.929	0.928	**0.932**
	**RMSE**	HGBR	10.882	10.012	9.141	9.402	8.706
		ETR	11.841	10.893	9.946	10.230	9.473
		DTR	10.007	9.207	8.406	8.646	8.006
		Average	10.91	10.04	9.16	9.43	**8.73**

## Methods

### Role and potential of ML in predictive processes

ML enables systems to learn patterns from data and make accurate predictions on new inputs without being explicitly programmed. The process begins with collecting and preprocessing high-quality data—handling missing values, normalizing features, and removing noise. During training, algorithms identify relationships within the data and iteratively adjust parameters to minimize prediction errors, with feature selection and engineering playing a crucial role in model accuracy. Once trained, ML models can generate various types of predictions and are widely applicable across fields such as forecasting, fraud detection, and recommendation systems. Despite its strengths, ML requires careful tuning to avoid issues like overfitting and bias. When properly managed, it delivers scalable, robust, and highly valuable predictive insights across many industries^24,25^.

### Utilized models and optimizers

The research harnesses synergy between ML regression models and optimization algorithms to improve prediction precision and minimize computational error in prediction procedures. Three regression models, namely Extra Trees Regression (ETR), Histogram Gradient Boosting Regression (HGBR), and Decision Tree Regression (DTR), were used. The models were then augmented using three optimization algorithms, including the Coyote Optimization Algorithm (COA) and the Osprey Optimization Algorithm (OOA). The synergy of these methodologies enables adaptive learning and stable model operation, especially with high-dimensional, non-linear, or noisy data.

**Fig. 2:**
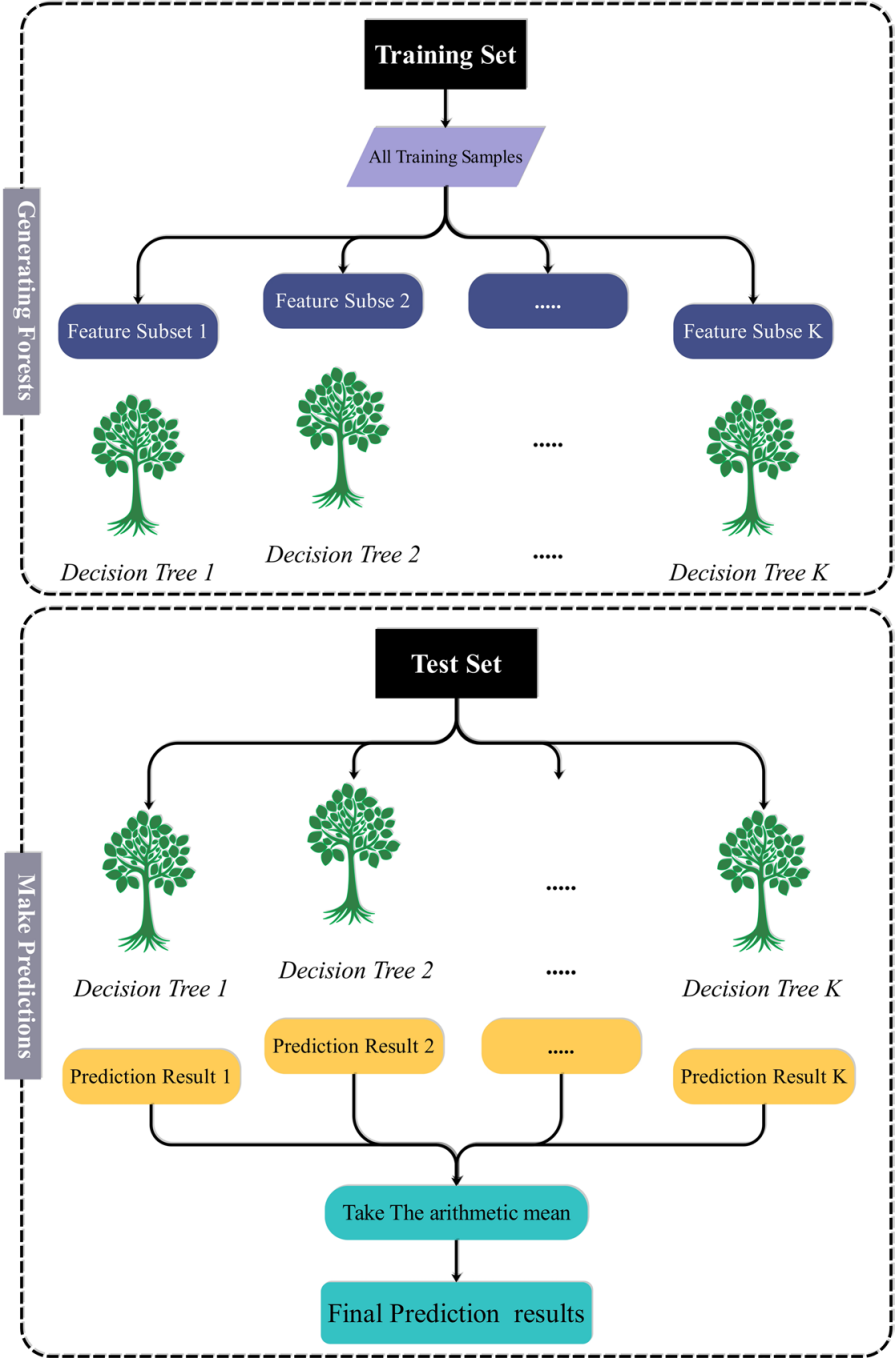
Structure of the ETR model.

ETR is an ensemble learning method based on random decision trees. Unlike standard decision tree-based models, ETR has randomness in not one but two phases: feature selection and split thresholds. Instead of finding the best split based on a fixed criterion like mean squared error reduction, ETR randomly picks split thresholds on a randomly selected subset of features at each node. The added randomness results in a larger reduction in variance and better generalization, particularly when the training set is complex or high-dimensional. As multiple trees are built independently and predictions are combined, often by averaging, the resulting prediction stabilizes and overfitting is reduced. The model is particularly suited to regression problems with noisy or unstructured data^26^. Figure 2 shows the flow chart of the ETR model.

**Fig. 3:**
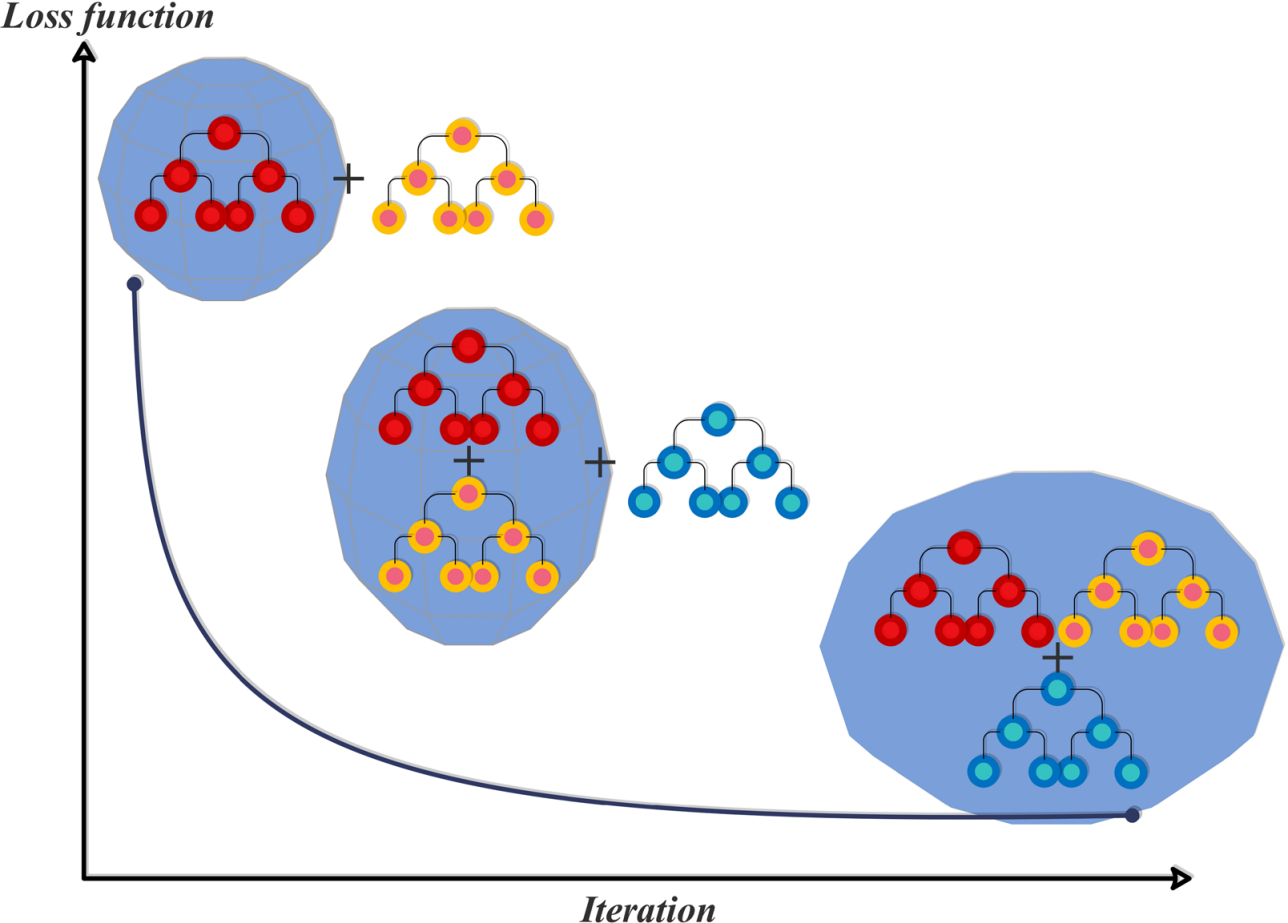
Structure of the HGBR model.

HGBR is a development of the standard gradient boosting algorithms. It constructs a forest of decision trees stage-wise, where each tree tries to reduce the residual errors of the current ensemble. The strength of HGBR lies in its efficiency: it converts continuous variables into integer-valued bins, accelerating training and minimizing memory consumption. Binning makes the model robustly scalable to large-scale data. HGBR also includes built-in regularization methods that control the complexity of the trees, balancing the model’s precision and its ability to generalize. It has built-in support for missing value handling and categorical feature encoding, making the preprocessing pipeline even simpler^27^. Figure 3 is the structure of the HGBR model.

**Fig. 4:**
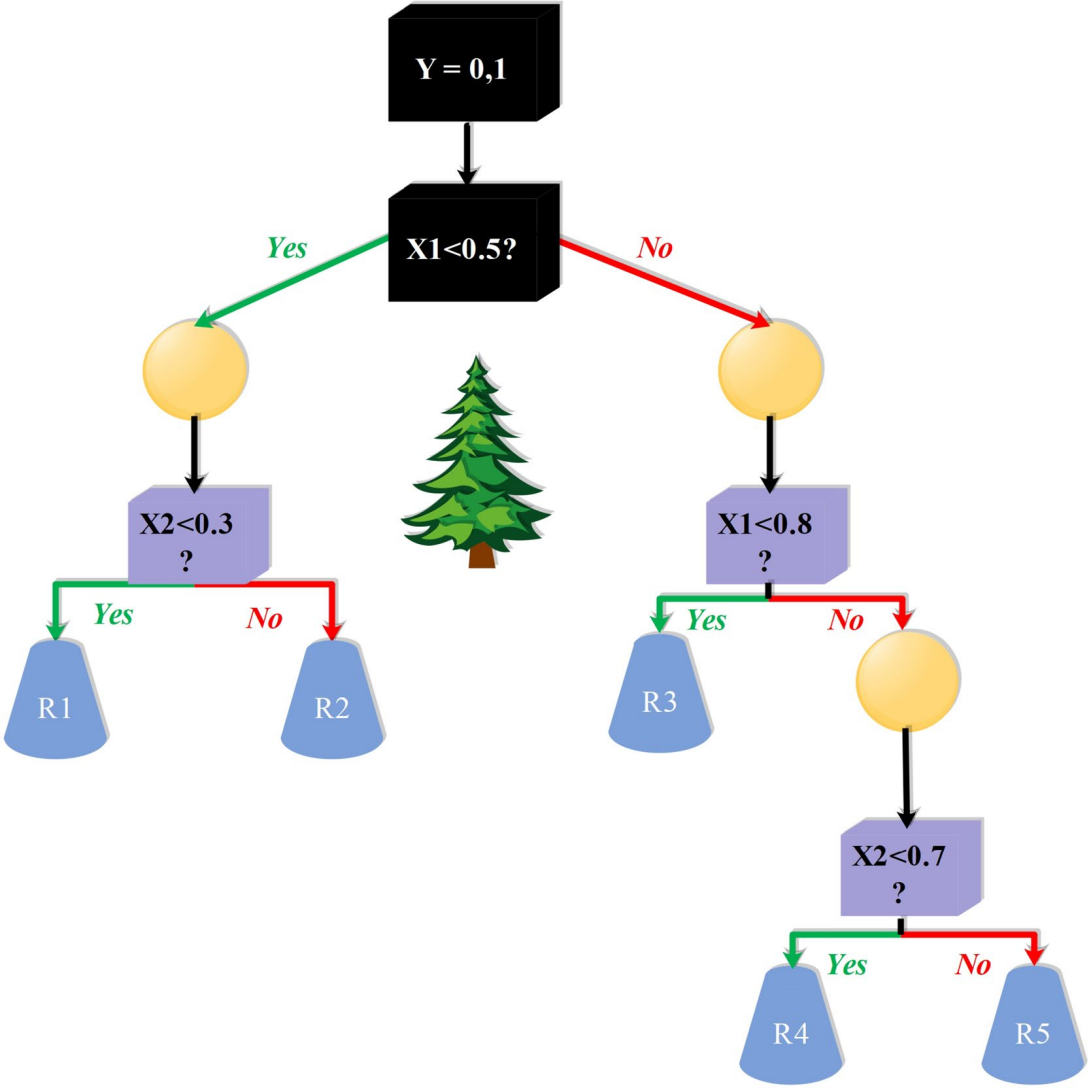
Structure of the DTR model.

DTR is a basic supervised learning algorithm that splits data into subsets based on values of the input feature and develops a tree structure where each node is a decision based on a single feature. The tree recursively splits the data to minimize the prediction error of the target variable, typically using criteria such as mean squared error. It is intuitive to understand and visualize, and it presents a simple graphical representation of decision paths. It tends to overfit, particularly when the tree becomes deep and picks up noise in the training data. As a single model, it performs well on problems with certain decision boundaries but is less robust on complex problems without regularization or ensemble combination^28^. Figure 4 shows the structure of the DTR model.

In order to overcome the limitations of single models and maximize predictive power, the models were coupled with sophisticated optimization algorithms. The COA is a population-based metaheuristic inspired by coyote social behavior. The COA models the learning and adaptive nature of coyote packs, in which solutions are improved through dynamic adaptation and interactions. Both exploration and exploitation are stressed by COA, balancing exploration of new regions of the solution space and fine-tuning of already known-quality solutions^29^.

**Fig. 5:**
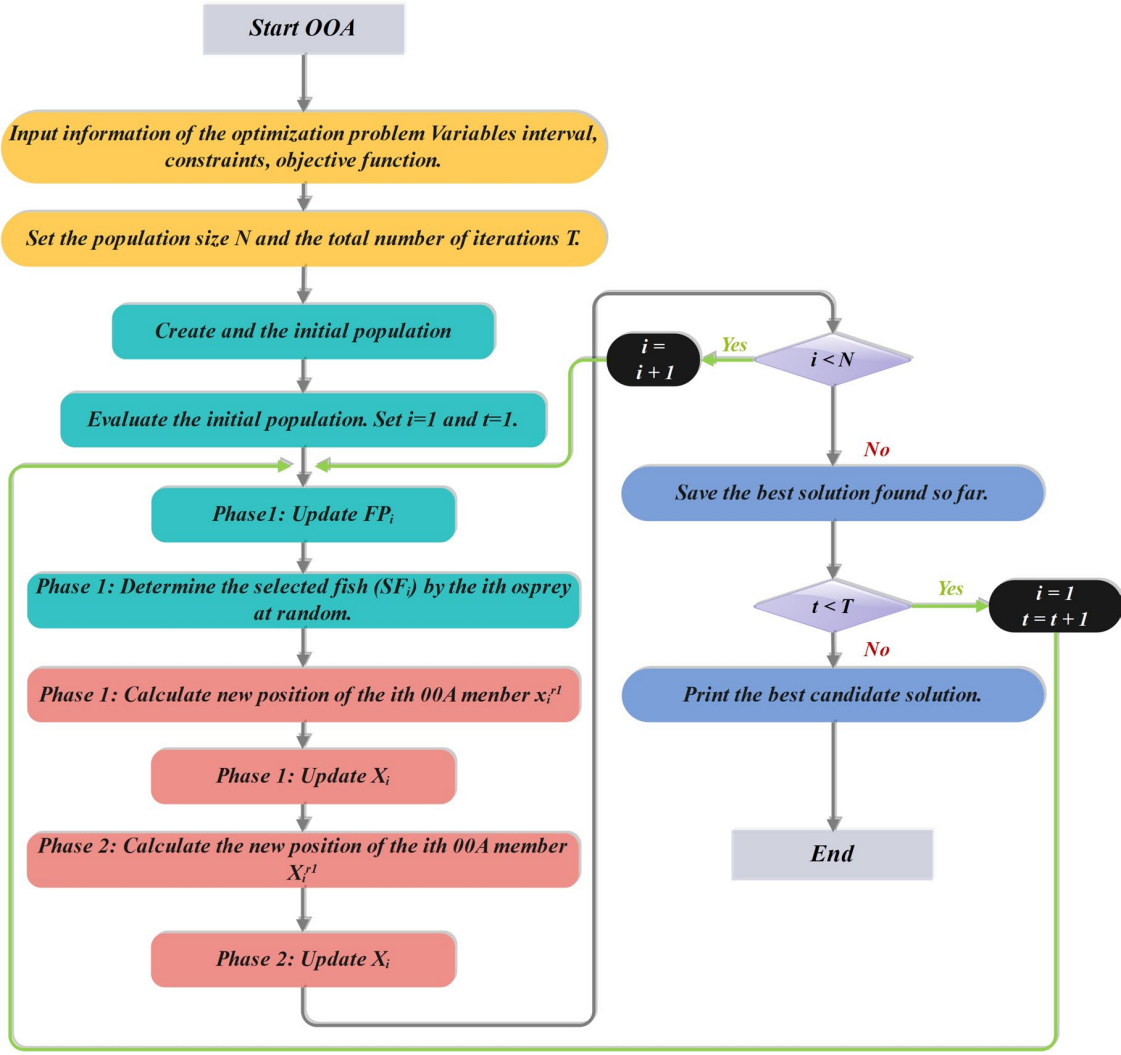
Flowchart of the OOA.

**Fig. 6:**
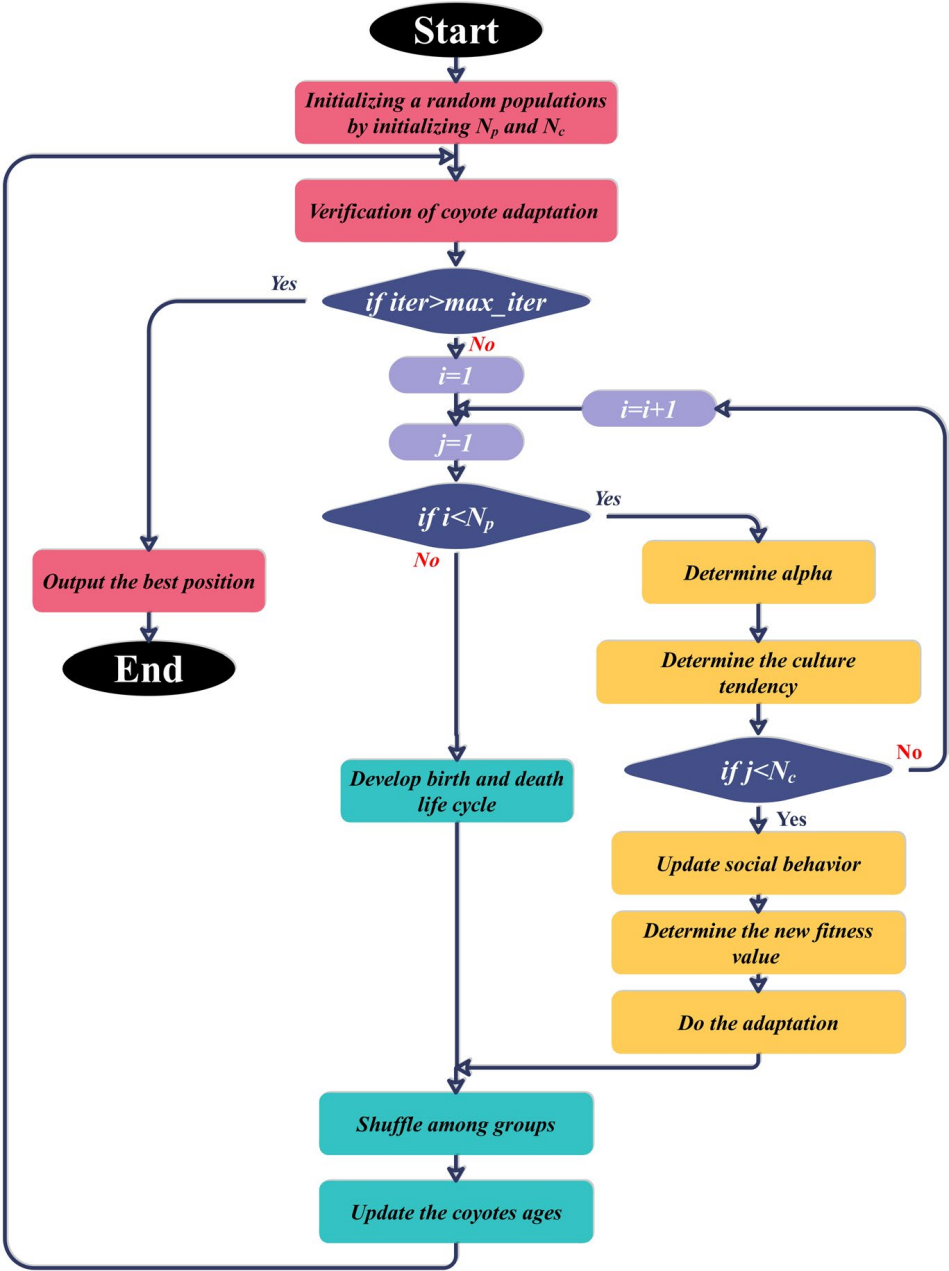
Flowchart of the COA algorithm.

The OOA is a bio-inspired algorithm based on Ospreys’ intelligent hunting tactics. The latest iteration enhances previously proposed versions by adding more dynamic control mechanisms, enabling real-time adaptation of the algorithm’s behavior to the optimization environment. Strategic hunting behavior is used by OOA to escape local optima and achieve faster convergence, making it suitable for tuning the parameters of intricate ML models^30^. Figures 5 and 6 show the flowcharts for the OOA and COA processes, respectively.

### Model and optimization algorithm selection

In this study, three tree-based regression models—HGBR, ETR, and DTR—were selected for their proven effectiveness in modeling complex, nonlinear relationships in energy-related datasets. Tree-based models offer several advantages, including the ability to handle heterogeneous data types, capture high-order feature interactions, and provide inherent feature importance measures, which are critical for interpretability in energy price and renewable energy forecasting.

The choice of three metaheuristic optimization algorithms, COA and OOA, was motivated by their demonstrated efficiency in navigating high-dimensional, non-convex search spaces, which is typical for hyperparameter tuning in tree-based models. These optimizers are particularly suited for improving model generalization and predictive performance by efficiently exploring complex hyperparameter landscapes.

While established methods such as Bayesian optimization, grid search, and genetic algorithms are widely used, they were not the focus of this study for two main reasons. First, grid search can become computationally prohibitive with high-dimensional hyperparameter spaces, particularly for large datasets (10,000 samples) with multiple tree-based models. Second, Bayesian optimization and genetic algorithms, though effective, often require extensive prior configuration and may not offer significant advantages over the selected metaheuristics in terms of convergence speed and solution diversity for this dataset type.

Overall, the combination of tree-based models with these three metaheuristic optimizers was chosen to balance predictive accuracy, computational efficiency, and interpretability, providing a robust framework for analyzing global energy price dynamics and renewable energy adoption.

### Performance evaluation metrics

This section presents the metrics used to evaluate the outcomes of the developed models.

1 $$\:{\mathrm{C}\mathrm{o}\mathrm{e}\mathrm{f}\mathrm{f}\mathrm{i}\mathrm{c}\mathrm{i}\mathrm{e}\mathrm{n}\mathrm{t}\:\mathrm{C}\mathrm{o}\mathrm{r}\mathrm{r}\mathrm{e}\mathrm{l}\mathrm{a}\mathrm{t}\mathrm{i}\mathrm{o}\mathrm{n}\:\left(\mathrm{R}2\right)R}^{2}={\left(\frac{{\sum\:}_{i=1}^{n}\left({b}_{i}-\stackrel{-}{b}\right)\left({m}_{i}-\stackrel{-}{m}\right)}{\sqrt{\left[{\sum\:}_{i=1}^{n}{\left({b}_{i}-\stackrel{-}{b}\right)}^{2}\right]\left[{\sum\:}_{i=1}^{n}{\left({m}_{i}-\stackrel{-}{m}\right)}^{2}\right]}}\right)}^{2}$$

2 $$\:\mathrm{R}\mathrm{o}\mathrm{o}\mathrm{t}\:\mathrm{M}\mathrm{e}\mathrm{a}\mathrm{n}\:\mathrm{S}\mathrm{q}\mathrm{u}\mathrm{a}\mathrm{r}\mathrm{e}\:\mathrm{E}\mathrm{r}\mathrm{r}\mathrm{o}\mathrm{r}\:\left(\mathrm{R}\mathrm{M}\mathrm{S}\mathrm{E}\right)RMSE=\sqrt{\frac{1}{n}{\sum\:}_{i=1}^{n}{\left({m}_{i}-{b}_{i}\right)}^{2}}$$

3 $$\:\mathrm{M}\mathrm{e}\mathrm{a}\mathrm{n}\:\mathrm{A}\mathrm{b}\mathrm{s}\mathrm{o}\mathrm{l}\mathrm{u}\mathrm{t}\mathrm{e}\:\mathrm{E}\mathrm{r}\mathrm{r}\mathrm{o}\mathrm{r}\:\left(\mathrm{M}\mathrm{A}\mathrm{E}\right)MAE=\frac{1}{n}\sum\:_{i=1}^{n}\left|{b}_{i}-{m}_{i}\right|$$

4 $$\:\mathrm{S}\mathrm{c}\mathrm{a}\mathrm{t}\mathrm{t}\mathrm{e}\mathrm{r}\:\mathrm{I}\mathrm{n}\mathrm{d}\mathrm{e}\mathrm{x}\:\left(\mathrm{S}\mathrm{I}\right)SI=\frac{RMSE}{mean\left({m}_{i}\right)}$$

5 $$\:\mathrm{P}\mathrm{r}\mathrm{e}\mathrm{d}\mathrm{i}\mathrm{c}\mathrm{t}\mathrm{i}\mathrm{o}\mathrm{n}\:\mathrm{i}\mathrm{n}\mathrm{t}\mathrm{e}\mathrm{r}\mathrm{v}\mathrm{a}\mathrm{l}\:\left(\mathrm{P}\mathrm{I}\right)\:PI={y}^{*}\pm\:\:t\times\:SE\times\:\sqrt{(1\:+\frac{1}{n}\:+\:\frac{{\left({x}^{*}-\stackrel{̄}{x}\right)}^{2}}{\varSigma\:{\left({x}_{i}-\stackrel{̄}{x}\right)}^{2}})}$$

The predicted value is represented by $\:{b}_{i}$, and $\:m̅$ and $\:b̅,$, respectively, stand for the measured and mean predicted values. The measured value is denoted by $\:{m}_{i}$. The value of the predictor variable for which the prediction is being made is represented by $\:{x}^{*}$. The mean of the predictor variable in the dataset is represented by $\:x̄$. $\:{y}^{*}$ is the predicted value.

### Hyperparameter tuning

The hyperparameter configurations for the HGBR-based hybrid models were obtained through a target-specific optimization process, reflecting the distinct characteristics of the Energy Price Index and Renewable Energy Share prediction tasks. The optimized settings demonstrate that the model structure and complexity must be tailored to the statistical properties of each target variable to achieve reliable predictive performance.

Table 3 summarizes the final hyperparameters obtained using the metaheuristic optimization algorithms (COA, OOA, and DDAO) for all developed hybrid models across both prediction targets. Notable variations in learning rate, tree depth, and leaf-related parameters indicate that each hybrid configuration required a distinct structural complexity to capture the underlying data patterns. For example, the HGBR-based hybrids predicting the Energy Price Index adopted moderately higher learning rates and larger numbers of leaf nodes, suggesting that more flexible tree structures were beneficial for modeling price variability. In contrast, models predicting Renewable Energy Share generally converged to lower learning rates and fewer leaf nodes, indicating a smoother, less volatile response surface.

A similar pattern is observed for the ETR- and DTR-based hybrid models, where the optimization process yielded substantially different values for maximum depth, number of estimators, and leaf constraints across targets and optimization algorithms. These variations highlight the sensitivity of tree-based methods to both hyperparameter scaling and data distribution.

All hyperparameter values reported in Table 3 correspond to the final solutions identified by the COA, OOA, and DDAO optimization procedures. A preliminary random search was used only to define reasonable parameter bounds prior to metaheuristic optimization and was not used for performance reporting or model comparison. This approach ensured efficient exploration of the hyperparameter space while maintaining methodological consistency and reproducibility.

**Table 3:** Hyperparameters of the developed models, along with their assigned values.

Target	Hyperparameter	Hybrid Models	
		HGCO	HGOA
*Energy Price Index (USD/kWh)*	learning_rate	0.154	0.119
	max_leaf_nodes	797	322
	max_depth	81	160
	min_samples_leaf	17	1
	max_bins	114	52
*Renewable Energy Share (%)*	learning_rate	0.032	0.43
	max_leaf_nodes	58	60
	max_depth	366	17
	min_samples_leaf	13	1
	max_bins	23	11
		***ETCO***	***ETOA***
*Energy Price Index (USD/kWh)*	n_estimators	139	384
	max_depth	135	151
	min_samples_split	0.001	0.001
	min_samples_leaf	0.0005	0.0005
	max_leaf_nodes	580	4450
*Renewable Energy Share (%)*	n_estimators	215	427
	max_depth	108	138
	min_samples_split	0.001	0.001
	min_samples_leaf	0.0005	0.0005
	max_leaf_nodes	1030	4120
		***DTCO***	***DTOA***
*Energy Price Index (USD/kWh)*	max_depth	140	999
	min_samples_split	0.00154 ‬‬‬‬	0.002 ‬‬‬‬
	min_samples_leaf	0.00077 ‬‬‬‬	0.0015 ‬‬‬‬
	max_leaf_nodes	348	2620
*Renewable Energy Share (%)*	max_depth	371	150
	min_samples_split	0.001	0.001
	min_samples_leaf	0.0005	0.0005
	max_leaf_nodes	920	5550

## Results

This section presents the empirical findings from ML regression models and their metaheuristic-optimized counterparts, evaluated across both the Energy Price Index and Renewable Energy Share prediction tasks. The analysis integrates performance metrics, convergence behaviors, and feature-level interpretability to assess the robustness and reliability of each optimized model. Comparative results highlight clear performance distinctions among the tested algorithms, revealing which hybrid approaches most effectively enhanced predictive accuracy. Additionally, incorporating explainability techniques—SHAP and CAM—provides deeper insight into the underlying drivers of model behavior, enabling a transparent examination of how key environmental and energy-related variables influence forecasting outcomes. Together, these results establish the foundation for understanding the relative strengths of the optimization strategies and the factors shaping global energy prediction dynamics.

**Fig. 7:**
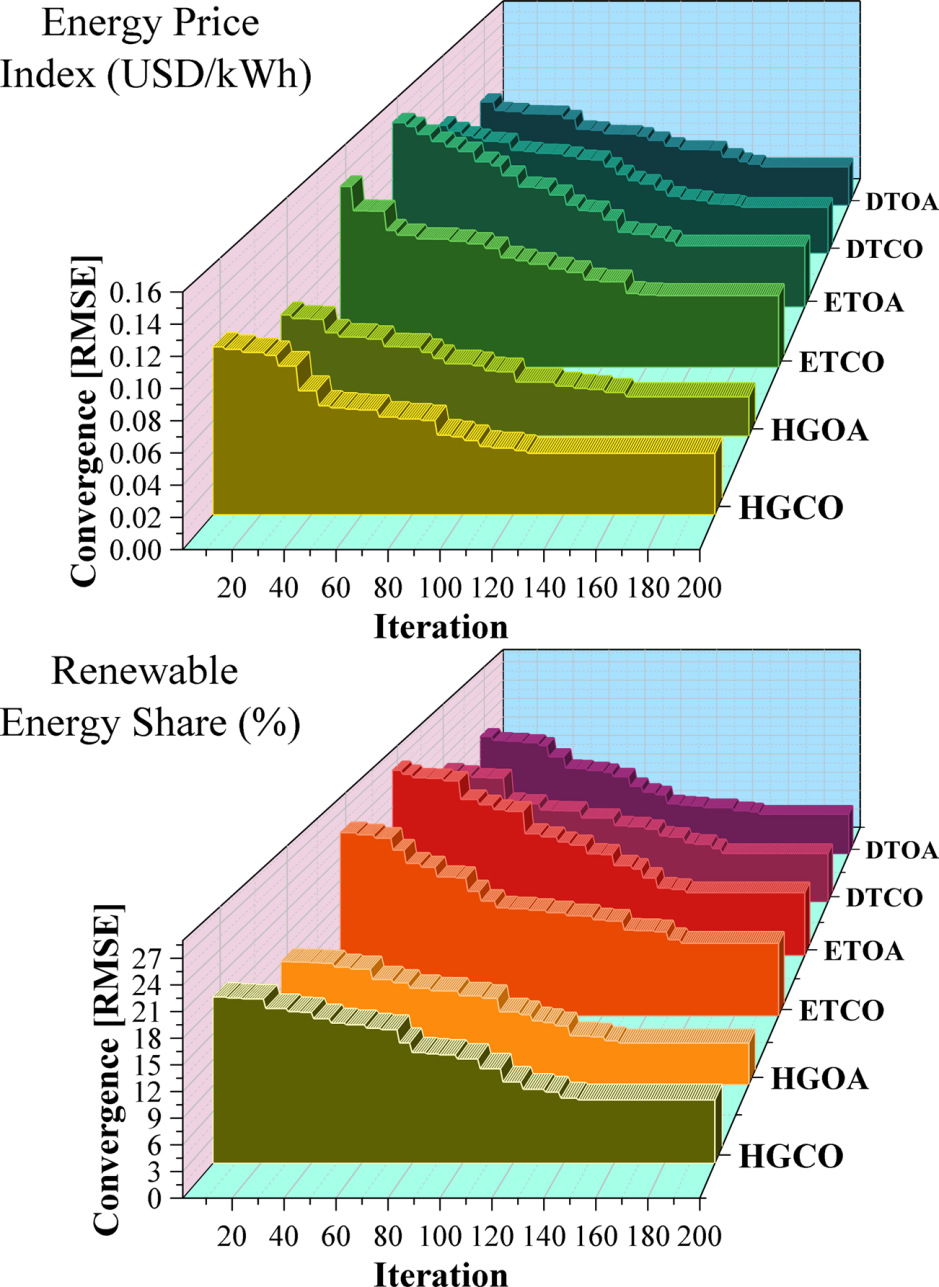
3D waterfall plot illustrating the convergence behavior of the optimization process across iterations or parameters.

Figure 7 indicates 3D waterfall plot for the convergence behavior of the optimization process across iterations or parameters. The evaluation compared several models using plots and tables to identify which achieved the best predictive accuracy. Across both scenarios—Energy Price Index (USD/kWh) and Renewable Energy Share (%)—the HGCO model consistently delivered the lowest RMSE and the strongest convergence, making it the top performer. HGOA showed stable and moderately good performance, but not at HGCO’s level. ETCO performed reasonably but with a higher RMSE, placing it in the mid-to-lower range. ETOA, DTCO, and especially DTOA showed weak convergence and high RMSE, marking them as the poorest performers. Overall, hybrid models like HGCO and HGOA proved more adaptable and accurate, while DTOA consistently ranked lowest.

The comparative evaluation of the developed models unfolded across the training, validation, and testing phases, employing statistical measures such as RMSE, R², MAE, SI, and PI to gauge predictive accuracy for the Energy Price Index in Table 4. Results revealed significant disparities in model effectiveness, underscoring variations in learning, generalization, and resilience. In the training phase, the HGOA hybrid model distinguished itself as the clear frontrunner. It achieved the lowest RMSE (0.0230) and MAE (0.0194) alongside the highest R² (0.9900), signifying outstanding fit and predictive reliability. While HGCO and DTOA also performed well, ETCO lagged among the hybrids, posting higher error metrics and diminished precision. Among single models, DTR was the most competitive, though it still could not match the hybrids’ performance. During validation, HGOA again demonstrated superior generalization, attaining the lowest RMSE (0.0345) and highest R² (0.9774). DTOA and HGCO maintained competitive performance, indicative of their stability. In contrast, ETCO recorded the weakest validation metrics, with the highest RMSE (0.0569) and lowest R² (0.9415) among hybrids. For single models, HGBR led, followed by ETR, while DTR had the lowest R² of 0.8963. Testing phase results remained consistent with prior phases: HGOA continued to excel, delivering the lowest RMSE (0.0341) and highest R² (0.9762), affirming its robust and reliable predictive capacity. DTOA and HGCO followed closely, while ETCO persisted as the weakest hybrid. Among single models, HGBR outperformed its peers, whereas DTR was again the least effective. Thus, HGOA consistently achieved outstanding performance across all phases and can be regarded as the most effective model in this context. DTOA and HGCO were also reliable, whereas ETCO fell short among hybrids. Of the single models, HGBR ranked highest, followed by ETR, with DTR showing the weakest predictive ability.

**Table 4:** Performance metrics for the models, evaluating their predictive capability and effectiveness in estimating the energy price index using key statistical measures.

*Target*	*Process*	Framework	Models	*Evaluation Metrics*				
				**RMSE**	**R** ^**2**^	**MAE**	**SI**	**PI**
***Energy Price Index (USD/kWh)***	***Training***	**Single**	HGBR	0.0455	0.9633	0.0385	0.1663	0.0840
			ETR	0.0401	0.9438	0.0328	0.1467	0.0744
			DTR	0.0379	0.9175	0.0305	0.1385	0.0708
		**Hybrid**	HGCO	0.0367	0.9788	0.0314	0.1344	0.0676
			HGOA	0.0230	0.9900	0.0194	0.0843	0.0422
			ETCO	0.0510	0.9601	0.0434	0.1865	0.0942
			ETOA	0.0458	0.9713	0.0391	0.1677	0.0845
			DTCO	0.0354	0.9364	0.0282	0.1295	0.0658
			DTOA	0.0308	0.9490	0.0243	0.1126	0.0570
	***Validating***	**Single**	HGBR	0.0517	0.9457	0.0437	0.1864	0.1864
			ETR	0.0457	0.9254	0.0374	0.1648	0.0840
			DTR	0.0433	0.8963	0.0352	0.0352	0.0803
		**Hybrid**	HGCO	0.0452	0.9634	0.0386	0.1631	0.0823
			HGOA	0.0345	0.9774	0.0293	0.1244	0.0626
			ETCO	0.0569	0.9415	0.0482	0.2053	0.1042
			ETOA	0.0523	0.9553	0.0442	0.1885	0.0953
			DTCO	0.0437	0.9170	0.0364	0.1575	0.0804
			DTOA	0.0391	0.9317	0.0324	0.1411	0.0718
	***Testing***	**Single**	HGBR	0.0506	0.9451	0.0431	0.1873	0.0949
			ETR	0.0467	0.9203	0.0383	0.1728	0.0882
			DTR	0.0424	0.8965	0.0348	0.1570	0.0806
		**Hybrid**	HGCO	0.0442	0.9629	0.0378	0.1636	0.0826
			HGOA	0.0341	0.9762	0.0290	0.1263	0.0635
			ETCO	0.0554	0.9430	0.0472	0.2049	0.1039
			ETOA	0.0509	0.9553	0.0435	0.1884	0.0953
			DTCO	0.0430	0.9166	0.0355	0.1592	0.0813
			DTOA	0.0390	0.9304	0.0321	0.1441	0.0734

Based on Table 5, when examining the performance of various models through the lens of SI and PI, it becomes evident which approaches yield reliable, robust predictions. To clarify, SI essentially measures the error relative to the variable’s range; a lower value indicates greater accuracy. PI serves as an indicator of the uncertainty inherent in the model’s predictions; lower values are preferable, reflecting greater confidence in the estimates. In forecasting the Energy Price Index, hybrid models consistently outperformed single models across all evaluation phases. The HGOA model, in particular, excelled during the training phase, delivering the lowest SI (0.0843) and PI (0.0422), both hallmarks of minimal error and high predictive certainty. DTOA and DTCO also produced commendable results, reinforcing their reliability. On the other hand, ETCO posted the highest SI (0.1865) and PI (0.0942) among the hybrids, which suggests notable weaknesses. During validation, HGOA continued to lead with the lowest SI (0.1244) and PI (0.0626), underscoring its ability to generalize. DTOA followed, maintaining solid metrics. ETCO and ETOA, however, continued to show elevated SI and PI values, likely due to overfitting or heightened sensitivity to data shifts.

For single models, DTR exhibited a strikingly low SI (0.0352) during validation, but this value appears anomalous when compared with RMSE and R², possibly a data reporting or formatting error. Testing phase results mirrored earlier findings: HGOA maintained its top position (SI = 0.1263, PI = 0.0635), with DTOA and DTCO trailing behind. ETCO remained the weakest among hybrids (SI = 0.2049, PI = 0.1039). Single models such as HGBR and DTR achieved moderate outcomes (SI = 0.157–0.187), but lagged behind their hybrid counterparts. Turning to Renewable Energy Share, HGOA again emerged as the leading model, recording the most favorable SI and PI across all phases. DTOA also performed effectively, particularly during the testing phase. Conversely, ETCO and ETOA persistently exhibited the highest SI and PI values, with ETCO’s figures in the testing phase (SI = 0.2214, PI = 0.1124) highlighting its instability. So, models characterized by lower SI and PI—especially HGOA—exhibited notable accuracy, consistency, and reliability. The findings underscore that hybrid models, particularly those enhanced by adaptive optimization techniques, offer substantial advantages in energy-related prediction tasks.

**Table 5:** Performance metrics for the models, evaluating their predictive capability and effectiveness in estimating renewable energy share using key statistical measures.

*Target*	*Process*	Framework	Models	*Evaluation Metrics*				
				**RMSE**	**R** ^**2**^	**MAE**	**SI**	**PI**
***Renewable Energy Share (%)***	***Training***	**Single**	HGBR	8.3555	0.9624	7.0087	0.1762	0.0889
			ETR	9.1335	0.9403	7.5662	0.1926	0.0978
			DTR	7.5363	0.9153	5.9791	0.1589	0.0812
		**Hybrid**	HGCO	7.0127	0.9751	5.9316	0.1479	0.0744
			HGOA	4.4820	0.9882	3.7508	0.0945	0.0474
			ETCO	9.3300	0.9593	7.8996	0.1967	0.0994
			ETOA	8.5259	0.9686	7.2266	0.1798	0.0906
			DTCO	6.7577	0.9335	5.3615	0.1425	0.0725
			DTOA	5.7147	0.9464	4.4577	0.1205	0.0611
	***Validating***	**Single**	HGBR	9.5702	0.9432	7.9634	0.2009	0.1019
			ETR	10.3283	0.9189	8.4833	0.2168	0.1107
			DTR	8.9794	0.8936	7.3954	0.1885	0.0969
		**Hybrid**	HGCO	8.1664	0.9630	6.9086	0.1714	0.0865
			HGOA	6.5528	0.9738	5.5307	0.1375	0.0692
			ETCO	10.3757	0.9415	8.7385	0.2178	0.1105
			ETOA	9.6775	0.9521	8.1445	0.2031	0.1028
			DTCO	8.1871	0.9149	6.7429	0.1719	0.0878
			DTOA	7.2942	0.9286	5.9849	0.1531	0.0780
	***Testing***	**Single**	HGBR	9.3752	0.9434	7.7950	0.2016	0.1023
			ETR	10.1102	0.9197	8.3559	0.2174	0.1110
			DTR	9.0347	0.8926	7.4703	0.1943	0.0999
		**Hybrid**	HGCO	7.9867	0.9632	6.7658	0.1717	0.0867
			HGOA	6.4258	0.9751	5.4549	0.1382	0.0695
			ETCO	10.2974	0.9394	8.6519	0.2214	0.1124
			ETOA	9.5474	0.9518	8.0126	0.2053	0.1039
			DTCO	8.2375	0.9141	6.8111	0.1771	0.0905
			DTOA	7.3591	0.9287	6.1239	0.1582	0.0806

**Fig. 8:**
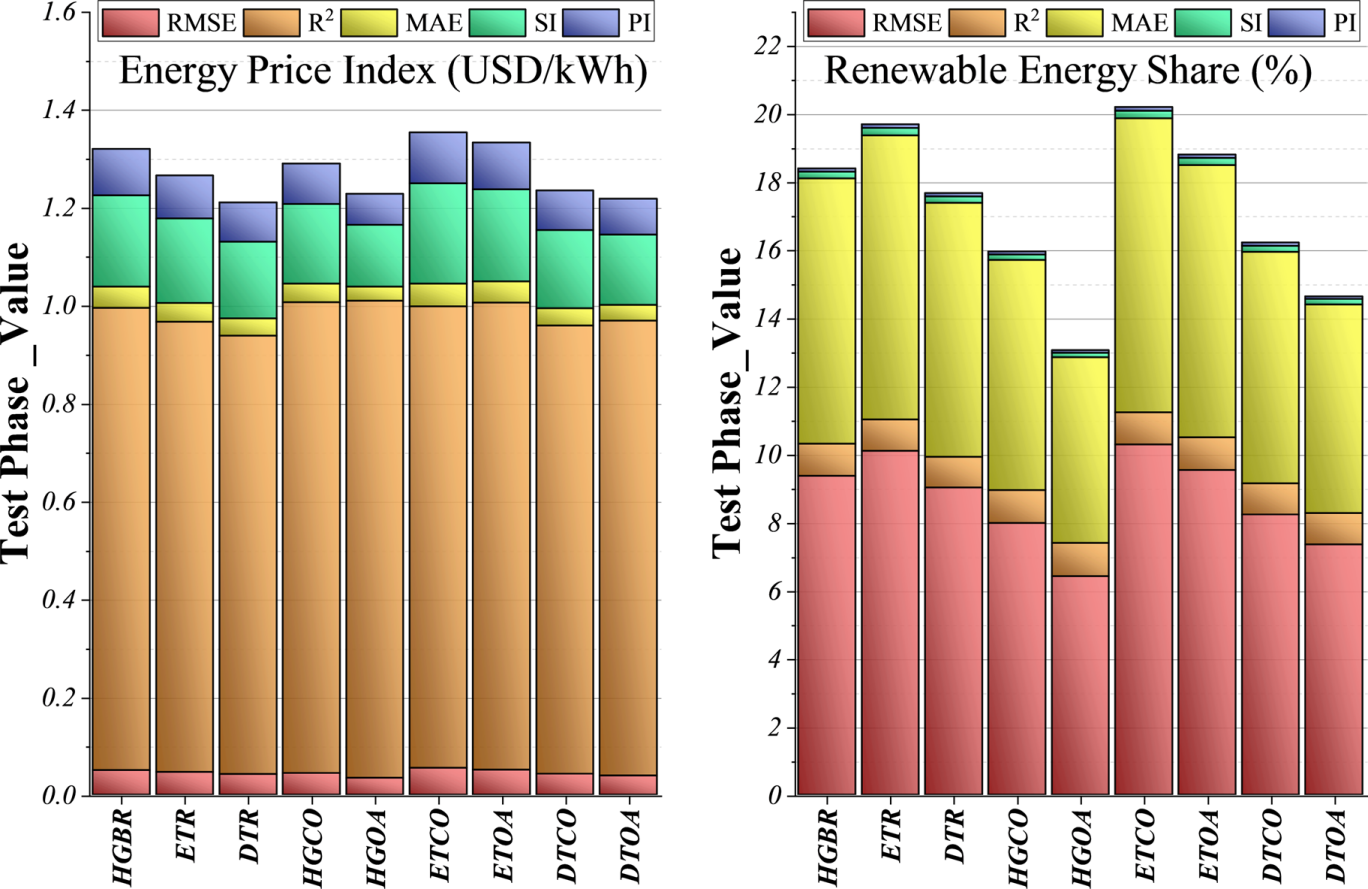
Stacked column plot comparing the performance metrics of different models, highlighting their accuracy and efficiency.

Figure 8 illustrates two column charts displaying model outcomes under two distinct scenarios: energy price and renewable energy share. Examining the renewable energy share condition first, the models contribute in a more distributed manner, one tends to form the base of the stacked bars, while others show varied input across the remaining segments. In contrast, under the energy price condition, one model dominates the lower segment consistently, with the other models’ contributions fluctuating above it, especially in the middle and upper portions of each bar. When comparing the two scenarios, the renewable energy share plot reflects a more balanced distribution among models, while the energy price plot indicates a stronger dependence on a single base model. The total column heights also fluctuate across cases, highlighting how some models adapt more effectively to different scenarios. These visual comparisons are valuable for assessing and enhancing predictive strategies. By observing how models perform differently under each condition, it becomes possible to identify which are most robust or specialized. Such insights enable more informed model selection, adjustment of ensemble strategies, and, ultimately, improved forecasting accuracy and reliability in energy-related contexts. Identifying these patterns supports the development of more resilient and context-aware predictive systems.

**Fig. 9:**
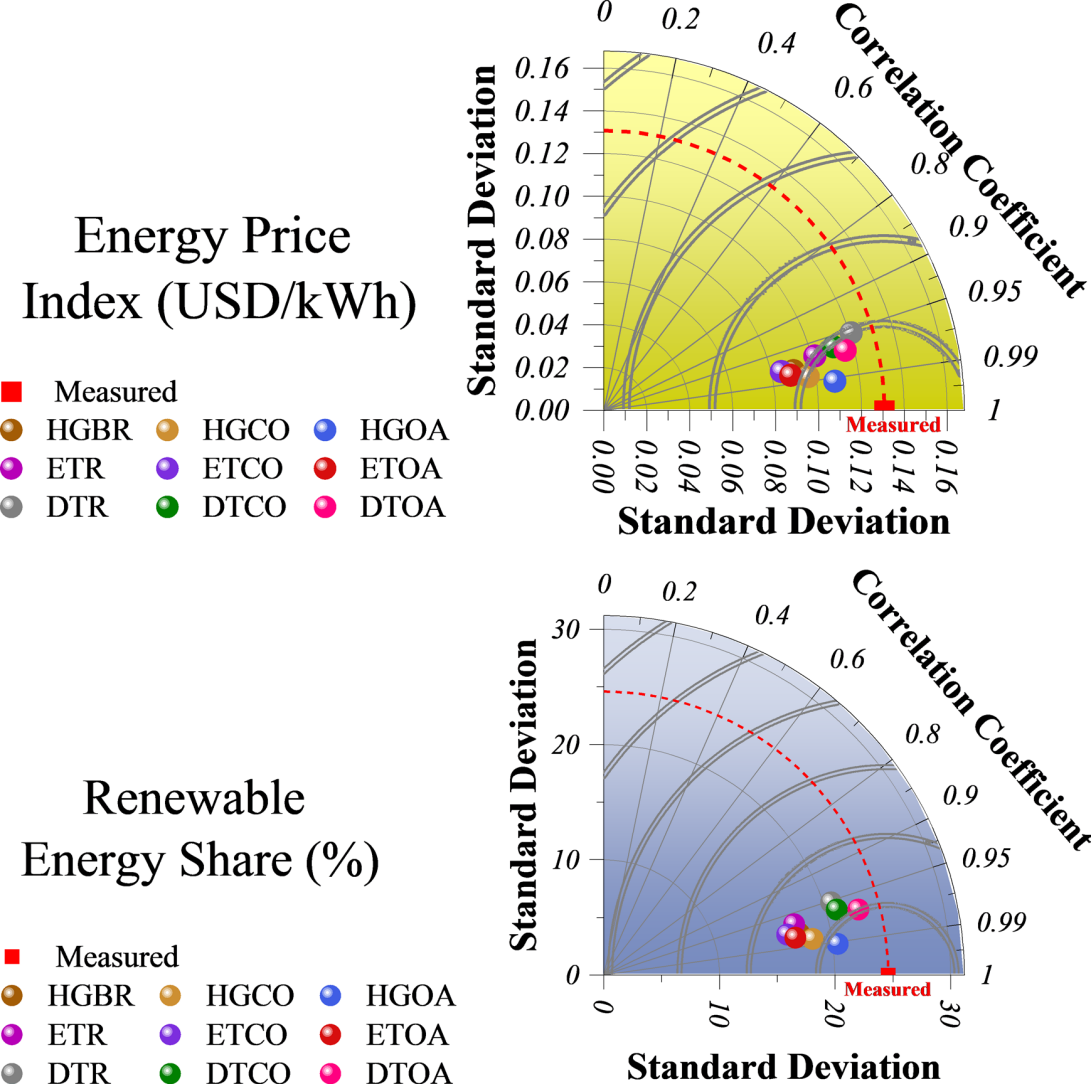
Taylor diagrams show the comparative performance of the models in estimating the Energy Price Index and the Renewable Energy Share, based on correlation coefficients and standard deviations.

The Taylor diagrams in Fig. 9 compare model predictions for the Energy Price Index and Renewable Energy Share by visualizing their correlation, standard deviation, and centered RMS error relative to observed data. For EPI, models such as HGOA and DTOA show the strongest alignment with measurements, as evidenced by their high correlations and proximity to the reference point. Models with larger standard deviations appear farther from the reference, indicating reduced precision. For RES, the models cluster more tightly around the reference point, reflecting generally higher correlations and better predictive reliability, with HGOA performing best and DTOA close behind. Models that remain near the reference across both diagrams are considered the most robust. However, varying standard deviations highlight uncertainties, suggesting that model suitability may differ depending on whether the focus is on market-driven energy prices or renewable energy adoption. Integrating multiple models and continually validating predictions against real-time data can enhance the reliability of forecasts for sustainable energy planning.

The statistical assessment of the leading models uncovers notable differences in standard deviation and skewness, highlighting how each model handles prediction spread and distribution in Table 6. For the Energy Price Index, the observed standard deviation is 0.12816, indicating moderate variation in the actual data. Among the models, DTOA demonstrates the greatest variability (0.104324), suggesting a broader dispersion of its prediction errors. In contrast, ETOA has the lowest standard deviation (0.083312), indicating more consistent, tightly clustered outputs. HGBR and HGOA fall between these extremes, with values of 0.084897 and 0.099037, respectively. Regarding skewness, the measured data are nearly symmetric (0.030425), while all models exhibit negative skew, reflecting a general tendency toward overestimation. ETOA’s skewness is the most pronounced (-0.51508), indicative of a strong left-tail bias, whereas DTOA is almost balanced (-0.02631). HGBR’s skewness (-0.44553) falls between these two, suggesting moderate left-skew. Shifting focus to Renewable Energy Share, the observed data present substantial variability, with a standard deviation of 24.631. DTOA again shows the highest variability among models (20.44407), capturing the wide range present in the actual data but also introducing greater prediction spread. ETOA, conversely, maintains the lowest dispersion (16.40994), reflecting higher consistency. Skewness across all models is slightly negative, with ETOA showing the most substantial left-skew (-0.523), indicating frequent overpredictions. DTOA, at -0.01966, closely aligns with the measured distribution. Based on the above explanations, ETOA produces the most consistent outputs across both targets, as evidenced by the lowest standard deviations, while DTOA most accurately reflects the distributional characteristics of the measured data, particularly in terms of skewness for Renewable Energy Share. The indicators reported in Table 6 are therefore interpreted as secondary consistency measures rather than as the primary basis for overall model ranking.

**Table 6:** Statistical metrics used to compare the top-performing models.

*Phase*	*Target*	*Models*	Properties					
			**Max**	**Min**	**Mean**	**Median**	**St. Dev**	**Skewness**
*Testing*	***Energy Price Index (USD/kWh)***	***Measured***	0.5	0.05	0.270	0.27	0.128	0.030
		***HGBR***	0.415	0.061	0.261	0.273	0.085	-0.446
		***HGOA***	0.453	0.061	0.267	0.272	0.099	-0.161
		***ETOA***	0.395	0.063	0.260	0.272	0.083	-0.515
		***DTOA***	0.492	0.06	0.269	0.272	0.104	-0.026
	***Renewable Energy Share (%)***	***Measured***	90	5.01	46.51	45.54	24.63	0.059
		***HGBR***	72.93	6.93	44.58	46.42	16.86	-0.473
		***HGOA***	81.18	6.91	45.70	46.18	19.27	-0.154
		***ETOA***	69.73	7.10	44.55	46.41	16.41	-0.523
		***DTOA***	88.40	6.26	46.27	46.44	20.44	-0.020

**Fig. 10:**
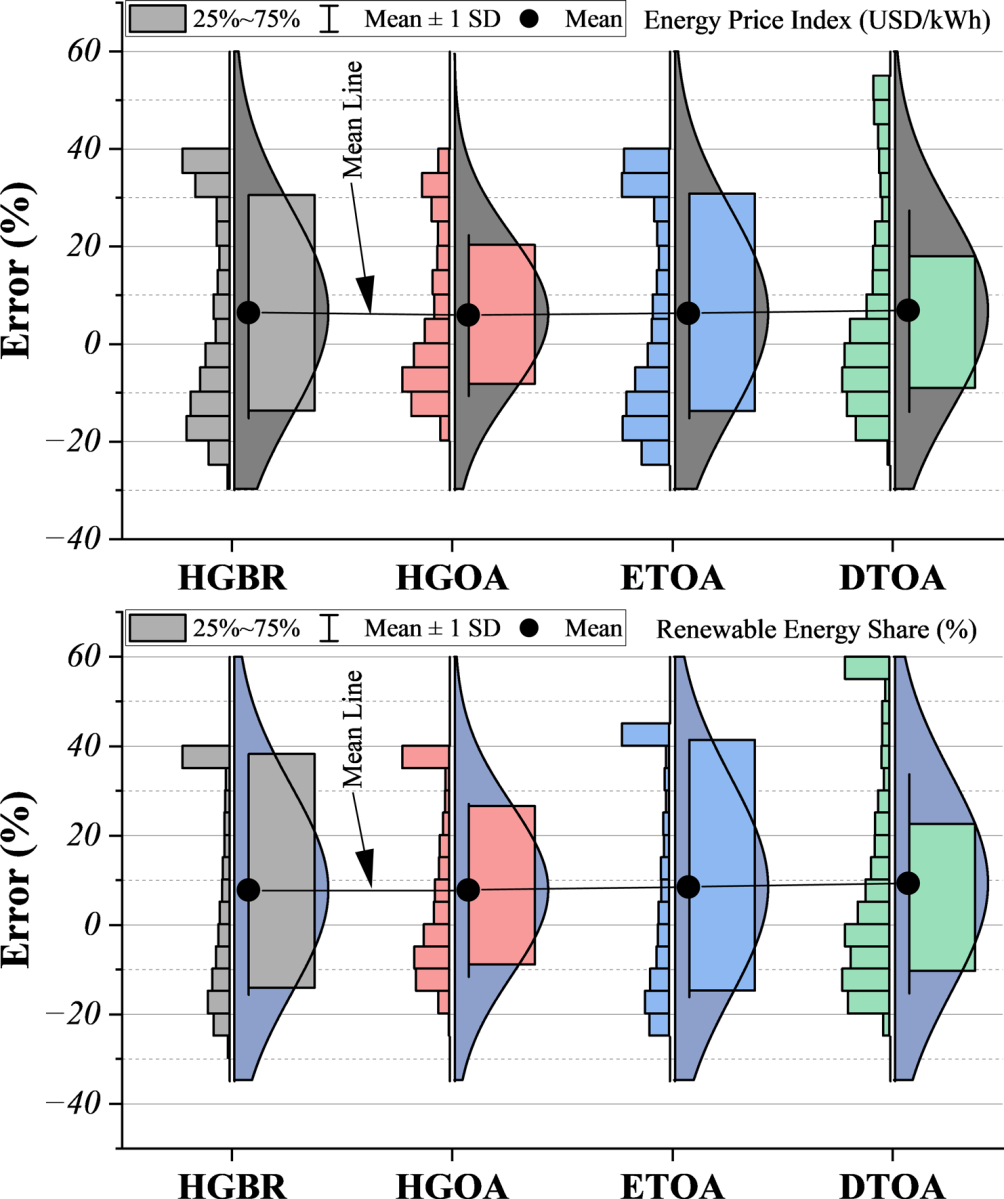
Half violin plot illustrating the error spread of the models’ predictions.

**Fig. 11:**
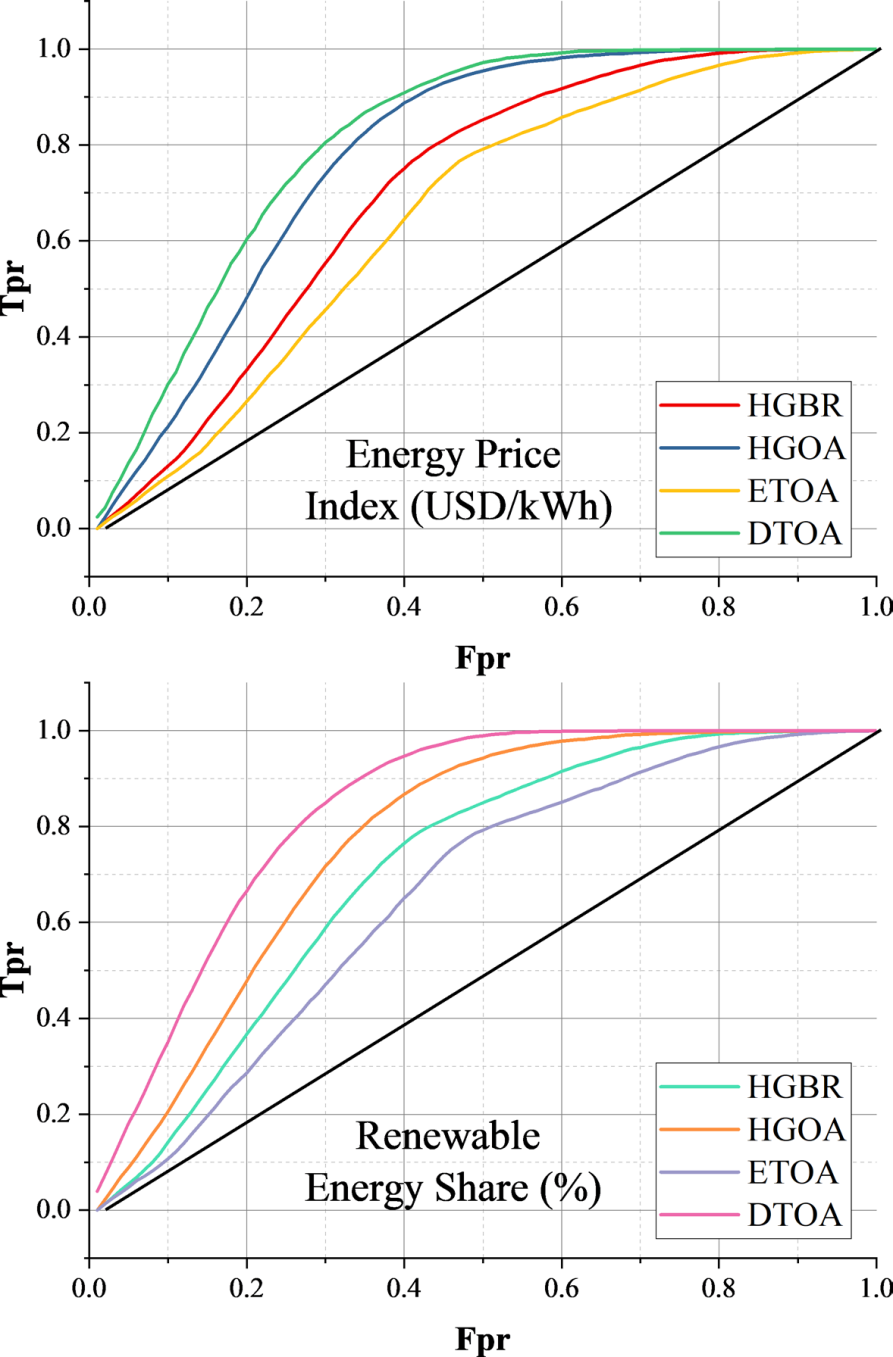
Line plot illustrating how prediction errors accumulate across models, with steeper REC curves indicating better accuracy and reliability.

The violin plots in Fig. 10 show that the DTOA model delivers the most reliable predictions, characterized by a tightly compressed error distribution, minimal outliers, and a median value near zero. These features indicate strong stability and low uncertainty. HGOA and ETOA demonstrate moderate performance, with comparatively narrower spreads and fewer outliers than HGBR, though they still exhibit greater variability than DTOA. HGBR performs the weakest, displaying a wide error range and numerous outliers, suggesting reduced predictive consistency. Although HGOA achieves the highest predictive accuracy for Renewable Energy Share according to RMSE and R² (Table 5), the DTOA configuration demonstrates greater robustness and stability across data partitions, as illustrated in Figs. 10 and 11.

Examining the line plots in Fig. 11 reveals how prediction errors accumulate across models. The REC curve’s steepness directly reflects a model’s predictive strength—a sharper incline suggests stronger accuracy and less error accumulation over time. DTOA stands out as the top performer, with the most pronounced and convex REC curve, implying consistently low error growth across the prediction horizon. HGOA also demonstrates strong predictive ability, closely following DTOA, though it does not quite reach the same level. ETOA is positioned in the middle: its performance is solid, though it neither excels nor underperforms compared to its peers. In contrast, HGBR’s rather flat REC curve indicates the weakest performance in this comparison, with higher error rates as predictions progress. Notably, the ranking of these models remains consistent across both plots: DTOA is the most reliable, followed by HGOA, then ETOA, with HGBR consistently lagging. For applications where minimizing prediction error is crucial, such as finance, supply chain management, and healthcare, DTOA is the most suitable option. HGOA and ETOA may be acceptable choices where some compromise between accuracy and computational efficiency is needed. HGBR, given its higher error accumulation, is less advisable for high-stakes or complex predictive tasks. In fact, employing REC curves to assess predictive models is not merely a theoretical exercise; it is a practical necessity. These curves offer valuable insight into model performance, enabling more informed and effective decisions regarding predictive analytics in real-world contexts.

**Fig. 12:**
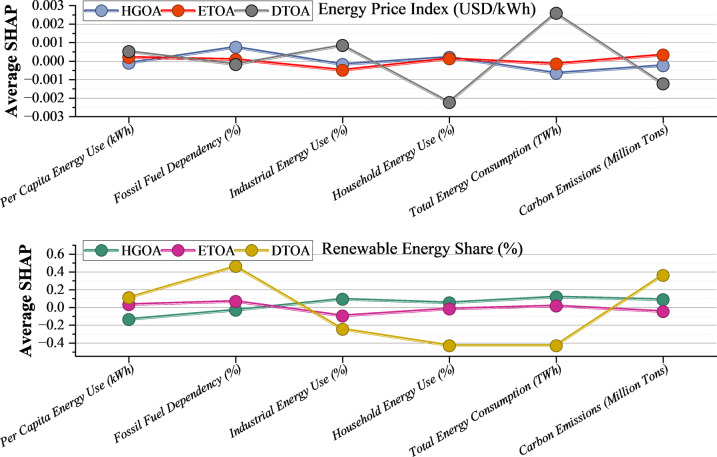
SHAP sensitivity analysis illustrating the contribution of each input feature to the model’s predictions.

Figure 12 provides a detailed view of how different variables influence the performance of the developed models. When examining the energy price index, it is clear that total energy consumption is the most significant driver for the DTOA model, whereas household energy consumption exhibits a comparatively minor effect. Conversely, in the ETOA and HGOA models, household energy use and carbon emissions are the most influential factors. When the focus shifts to the share of renewable energy, the DTOA model becomes highly sensitive to both fossil fuel dependency and carbon emissions. In contrast, ETOA and HGOA models are most affected by total energy consumption and industrial energy use. The pronounced impact of fossil fuel dependency, total energy consumption, and carbon emissions on the accuracy of global energy consumption predictions stems from the interconnected roles these variables play within energy systems. Fossil fuel dependency, defined by the proportion of non-renewable sources in the energy mix, significantly shapes predictions due to its predominance in global supply chains and susceptibility to geopolitical or economic disruptions. Fluctuations in fossil fuel markets can directly shift consumption patterns, requiring models to adapt dynamically to these changes. Total energy consumption serves as a fundamental indicator, encapsulating demand across industrial, residential, and commercial sectors. It is closely correlated with economic activity, population growth, and urbanization, making it a central variable for forecasting. However, disaggregating this data to reflect sector-specific impacts remains a methodological challenge. Carbon emissions act as both a proxy for fossil fuel usage and an indicator of environmental policy effectiveness. Changes in regulatory frameworks or carbon pricing mechanisms can alter consumption trajectories, prompting shifts toward renewable sources and necessitating complex adjustments in predictive models. The interactions between these variables are often non-linear, demanding advanced modeling approaches, such as ML, to effectively capture feedback mechanisms and temporal dependencies. Uncertainties in data quality, such as incomplete emission inventories or inconsistent consumption reporting, can undermine predictive reliability. Integrating these variables enables models to forecast global energy consumption better, but the inherent complexity requires continuous refinement to remain responsive to evolving energy trends and policy landscapes.

**Fig. 13:**
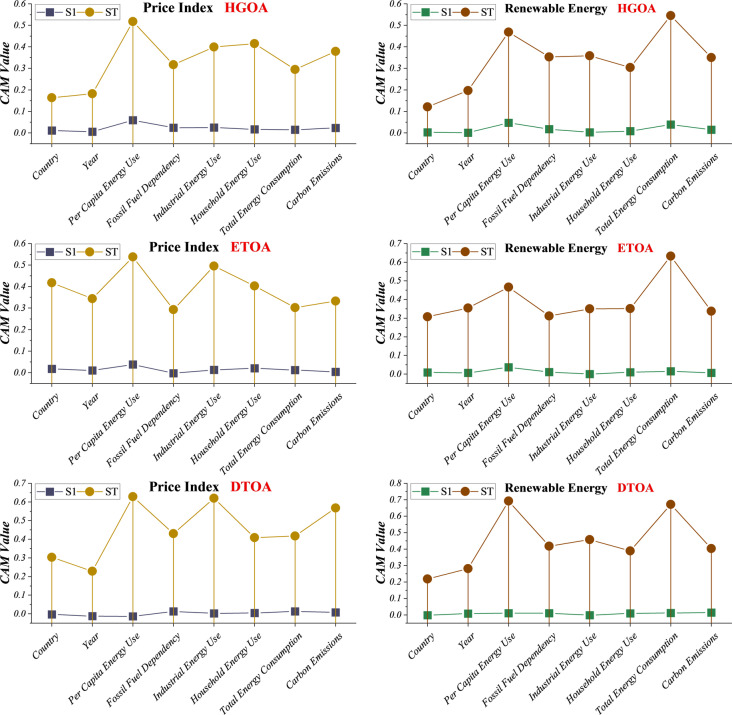
CAM sensitivity analyses for the impact of the input variables on first-order and total index sensitivity.

Figure 13 presents the CAM-based sensitivity analyses for the Price Index and Renewable Energy Share under the HGOA, ETOA, and DTOA models, highlighting how each input variable contributes to both first-order (S1) and total-order (ST) sensitivity. Across all models and targets, the ST values are consistently much higher than the S1 values, indicating substantial interaction effects among variables rather than isolated, single-variable influence. For both the Price Index and Renewable Energy predictions, Per Capita Energy Use, Fossil Fuel Dependency, and Household Energy Use are the most influential factors, as reflected in their dominant ST values. Carbon emissions and country/year identifiers show relatively low sensitivity, suggesting a weaker direct influence on model outputs. Differences among models are also evident. HGOA exhibits the greatest variability in ST values, suggesting stronger interaction-driven behavior. ETOA shows a more moderate, balanced sensitivity pattern, whereas DTOA demonstrates a sharply defined set of dominant variables, reflecting its stable, focused predictive structure. Overall, the figure confirms that energy consumption intensity and fossil fuel reliance are the primary drivers in both pricing and renewable energy forecasting, and it highlights the importance of variable interactions in shaping model behavior.

In addition, Table 7 presents the second-order sensitivity indices (S2) for paired input variables across the Price Index and Renewable Energy targets for the HGOA, ETOA, and DTOA models. The S2 index quantifies interaction effects between two variables, revealing how combinations of inputs affect model outputs beyond their individual contributions. Notably, variable interactions differ across models. In HGOA, strong interactions are observed between Per Capita Energy Use and Carbon Emissions, Per Capita Energy Use and Household Energy Use, and Industrial Energy Use with Total Energy Consumption, particularly in predicting the Price Index. DTOA exhibits smaller or even negative S2 values, indicating weaker interactive effects or compensatory influences, especially in Renewable Energy forecasts. ETOA shows mixed behavior, with some variable pairs, such as Year and Total Energy Consumption, producing moderate positive interactions for Renewable Energy. Across all models, energy consumption metrics—Per Capita, Industrial, Household, and Total Energy Use—combined with either Carbon Emissions or Fossil Fuel Dependency consistently yield the largest S2 values. This highlights the critical role of interdependent energy and environmental factors in forecasting. In contrast, interactions involving Country and Year typically exhibit lower S2 values, suggesting that spatiotemporal identifiers primarily contribute through additive rather than interactive effects. Target-specific patterns are also evident. For the Price Index, interaction effects are more pronounced, especially in HGOA and DTOA, indicating that energy pricing is highly sensitive to multi-variable interactions. For Renewable Energy, S2 values are generally smaller or more balanced, suggesting that renewable adoption may depend more on dominant single-variable effects than on complex interactions.

**Table 7:** CAM results for paired input variables across the price index and renewable energy targets.

Variable	Integrated with	Parameter					
		S2					
		Price Index			Renewable Energy		
		HGOA	ETOA	DTOA	HGOA	ETOA	DTOA
Country	Year	0.0086	0.0138	0.0049	-0.0029	-0.0041	-0.0088
Country	Per Capita Energy Use	0.0048	0.0202	0.0239	0.0011	-0.0060	0.0116
Country	Fossil Fuel Dependency	0.0098	0.0039	-0.0070	-0.0048	-0.0071	0.0050
Country	Industrial Energy Use	0.0091	0.0076	0.0016	0.0000	0.0006	-0.0023
Country	Household Energy Use	0.0060	0.0196	-0.0051	-0.0077	-0.0046	-0.0081
Country	Total Energy Consumption	0.0053	0.0050	-0.0005	-0.0050	0.0219	0.0130
Country	Carbon Emissions	0.0139	0.0120	0.0046	0.0047	-0.0055	-0.0027
Year	Per Capita Energy Use	0.0032	-0.0029	0.0136	0.0177	0.0040	0.0109
Year	Fossil Fuel Dependency	-0.0018	0.0019	0.0179	0.0111	-0.0025	-0.0133
Year	Industrial Energy Use	0.0073	0.0109	0.0182	0.0137	0.0117	0.0073
Year	Household Energy Use	0.0095	0.0041	0.0075	0.0113	0.0111	-0.0029
Year	Total Energy Consumption	0.0070	0.0081	0.0124	0.0161	0.0252	0.0006
Year	Carbon Emissions	0.0049	0.0072	0.0232	0.0117	0.0087	-0.0156
Per Capita Energy Use	Fossil Fuel Dependency	0.0098	0.0023	0.0026	0.0143	0.0012	-0.0023
Per Capita Energy Use	Industrial Energy Use	0.0240	0.0063	0.0205	0.0127	0.0024	-0.0021
Per Capita Energy Use	Household Energy Use	0.0297	-0.0049	0.0064	-0.0041	-0.0123	-0.0047
Per Capita Energy Use	Total Energy Consumption	0.0081	-0.0108	0.0099	0.0437	0.0418	0.0243
Per Capita Energy Use	Carbon Emissions	0.0342	0.0089	0.0044	0.0106	0.0031	-0.0124
Fossil Fuel Dependency	Industrial Energy Use	0.0101	0.0193	-0.0186	0.0250	0.0168	-0.0144
Fossil Fuel Dependency	Household Energy Use	0.0086	0.0144	-0.0131	0.0074	0.0091	0.0056
Fossil Fuel Dependency	Total Energy Consumption	0.0056	0.0118	-0.0277	0.0161	0.0178	0.0131
Fossil Fuel Dependency	Carbon Emissions	-0.0006	0.0224	-0.0179	0.0116	0.0067	0.0109
Industrial Energy Use	Household Energy Use	0.0199	0.0067	-0.0046	0.0089	0.0128	0.0009
Industrial Energy Use	Total Energy Consumption	0.0231	0.0056	-0.0001	0.0284	0.0383	0.0183
Industrial Energy Use	Carbon Emissions	0.0228	0.0070	0.0093	0.0113	0.0152	0.0007
Household Energy Use	Total Energy Consumption	0.0337	0.0030	-0.0168	0.0099	0.0099	-0.0028
Household Energy Use	Carbon Emissions	0.0230	-0.0051	0.0009	0.0155	0.0052	-0.0009
Total Energy Consumption	Carbon Emissions	0.0077	0.0005	-0.0169	0.0115	0.0291	-0.0027

## Discussion

### Application and real-world implications based on feature analyses

The findings from CAM, SHAP, and second-order sensitivity analyses provide actionable insights for energy policy, market planning, and renewable energy deployment. The identification of key drivers—such as total energy consumption, household and industrial energy use, fossil fuel dependency, and carbon emissions—highlights the variables that most strongly influence energy prices and renewable energy adoption. Policymakers can use this information to target interventions effectively. For example, reducing fossil fuel dependency or incentivizing efficient household and industrial energy use can directly impact both energy costs and the share of renewables in the energy mix.

The pronounced interaction effects revealed in HGOA suggest that policies must account for interdependencies among variables. A change in one factor, such as carbon pricing or fossil fuel supply disruptions, can propagate through the system, affecting consumption patterns, energy costs, and renewable adoption simultaneously. Recognizing these non-linear effects allows governments and energy planners to anticipate unintended consequences of interventions, such as market volatility or delayed adoption of renewable technologies. For energy markets, understanding the dominant predictors of the Price Index can enhance forecasting accuracy, enabling more informed investment decisions, risk management, and tariff design. Energy companies can prioritize infrastructure upgrades, storage capacity, and demand-side management programs based on variables that have the greatest influence on energy prices.

In the renewable energy domain, insights from DTOA, ETOA, and HGOA suggest that scaling up renewable capacity is most sensitive to fossil fuel dependency and overall energy demand. This underscores the importance of integrating renewable planning with broader energy system reforms, including grid modernization, energy efficiency programs, and diversification of energy sources. Finally, the robust identification of key variables and their interactions facilitates the development of adaptive, data-driven policies. By continuously monitoring these factors and updating models in response to real-world changes, governments and organizations can design flexible strategies that balance economic growth, environmental sustainability, and energy security. Overall, these insights bridge the gap between predictive modeling and actionable policy, enabling informed decision-making in complex energy systems.

### From accuracy to value-oriented forecasting

The predictive framework proposed in this study is fundamentally accuracy-oriented, with model training and evaluation guided by minimizing prediction error using metrics such as RMSE and R². This design choice reflects the primary objective of delivering reliable, interpretable forecasts of the Energy Price Index and Renewable Energy Share, which serve as essential informational inputs for energy policy assessment, market monitoring, and strategic planning. In such applications, reducing statistical forecast error remains a necessary prerequisite for informed analysis and scenario evaluation.

In recent years, however, the forecasting literature has increasingly emphasized value-oriented prediction paradigms, which aim to generate forecasts that directly enhance downstream decision outcomes rather than solely optimizing traditional accuracy metrics. These approaches are often formulated within closed-loop predict-then-optimize frameworks, where predictive models are embedded into operational or economic decision processes and iteratively refined to maximize decision value (e.g., economic benefit or operational efficiency) rather than predictive accuracy alone^31,32^. Such studies demonstrate that forecasts with comparable or even slightly inferior error metrics may yield superior real-world performance when evaluated from a decision-centric perspective.

While the present study does not explicitly implement a value-oriented or closed-loop optimization layer, the proposed framework is naturally compatible with such extensions. The optimized tree-based models provide accurate, stable, and explainable predictions that can serve as high-quality inputs to downstream optimization tasks, including energy portfolio allocation, capacity expansion planning, and policy intervention design. From this perspective, the current work establishes a robust and interpretable prediction module that can be integrated into future predict-then-optimize systems.

Consequently, extending the proposed framework toward value-oriented forecasting and closed-loop decision-making settings represents a promising avenue for future research. Such extensions would enable direct assessment of how improvements in predictive accuracy and interpretability translate into measurable economic, operational, or policy value within energy systems.

## Conclusion

This research pursued two principal objectives. The first was to predict global energy consumption using several Machine Learning (ML) regression techniques, including Extra Trees Regression, Histogram Gradient Boosting Regression (HGBR), and Decision Tree Regression (DTR). These models were further refined using advanced optimization algorithms, namely the Coyote Optimization Algorithm (COA) and Osprey Optimization Algorithm (OOA), to enhance their predictive accuracy for energy price indices and renewable energy shares. The second and primary objective was to identify the key factors influencing model performance using SHAP (SHapley Additive exPlanations) and Cosine Amplitude Method (CAM) sensitivity analysis. The findings revealed that, in predicting the share of renewable energy, fossil fuel dependency and carbon emissions exerted the most substantial influence, highlighting their critical role in the performance of the best-modified DTOA model. Conversely, for predicting the energy price index, total energy consumption emerged as the predominant factor affecting DTOA performance, emphasizing the model’s sensitivity to aggregate demand across industrial, residential, and commercial sectors. These results underscore the importance of energy system interdependencies and suggest that targeted interventions, such as reducing reliance on fossil fuels to accelerate renewable adoption or managing overall consumption to stabilize energy prices, could have meaningful impacts on energy planning and policy design. Moreover, the study demonstrated that advanced ML models, when combined with optimization techniques, could capture complex, non-linear relationships among energy consumption patterns, carbon emissions, fossil fuel dependency, and other key variables, thereby improving forecasting reliability. The feature analyses using SHAP and CAM confirmed the dominant contributions of energy consumption intensity, fossil fuel dependency, and carbon emissions, while also highlighting interactions among these variables. These insights not only reinforced the models’ interpretability but also provided actionable guidance for decision-makers seeking to design adaptive, data-driven strategies that balance energy security, economic growth, and environmental sustainability. Overall, the research established a robust framework for both predictive accuracy and interpretive understanding in global energy modeling.

## Data Availability

Data will be provided upon request to the corresponding author.
